# State of the Art of Family Quality of Life in Early Care and Disability: A Systematic Review

**DOI:** 10.3390/ijerph17197220

**Published:** 2020-10-02

**Authors:** Carmen Francisco Mora, Alba Ibáñez, Anna Balcells-Balcells

**Affiliations:** 1 Faculty of Psychology, Education and Sports Sciences, Ramon Llull University, 08022 Barcelona, Spain; carmenrm@blanquerna.url.edu (C.F.M.); annabb0@blanquerna.url.edu (A.B.-B.); 2 Faculty of Education, University of Cantabria, 39005 Santander, Spain; 3 Group in Health Economics and Management of Health Services, Instituto de Investigación Sanitaria Valdecilla (IDIVAL), 39011 Santander, Spain

**Keywords:** family quality of life, disability, early childhood intervention, conceptualization, measurement

## Abstract

*Background*: In recent years, there has been a growing international interest in family quality of life The objective of this systematic review is to understand and analyze the conceptualization of the quality of life of families with children with disabilities between 0 and 6 years of age, the instruments for their measurement and the most relevant research results. *Method*: A bibliographic search was conducted in the Web of Science, Scopus and Eric databases of studies published in English and Spanish from 2000 to July 2019 focused on “family quality of life” or “quality of family life” in the disability field. A total of 63 studies were selected from a total of 1119 and analyzed for their theoretical and applied contributions to the field of early care. *Results*: The functional conceptualization of family quality of life predominates in this area, and a nascent and enriching holistic conceptualization is appreciated. There are three instruments that measure family quality of life in early care, although none of them is based on unified theory of FQoL; none of them focus exclusively on the age range 0–6 nor do they cover all disabilities. *Conclusions*: The need to deepen the dynamic interaction of family relationships and to understand the ethical requirement that the methods used to approach family quality of life respect the holistic nature of the research is noted.

## 1. Introduction

The field of early care (EC) is currently undergoing a significant conceptual change. The former clinical intervention model (expert model) is now being replaced by a social and transdisciplinary model (collaboration model) in which family and the environment are core dimensions [1,2]. Development studies have acknowledged the relevance of the social and cultural nature of human development since birth [3,4]. Yet, the role that the people surrounding the children and interacting with them from their birth and throughout their development process has been underestimated [5,6,7,8,9].

The focus now is on a positive understanding of disability and a knowledge “of the capacity for positive adaptation and of the strengths of families with children with disabilities” [10] (p. 2). Considering the positive impact that the families have in the development of kids with disabilities, the strategy now is to work with the families rather than working for the families [11], and to involve all the family members because what happens to any of the members has an impact on the rest of the family. Thus, it is important to take into consideration the individual needs of each of the family members as well as the needs of the family as a whole [12]. 

The new focus of EC on the family contributes to overcome some of the limitations that the ambulatory model had, especially regarding the time that the child can engage in learning opportunities and the type of learning opportunities that the family context offers [13]. The family involvement in turn fosters greater parental responsibility, improves family skills, and generates a higher level of satisfaction in the family [14]. Moreover, as Samuel et al., argue, “families that function well support societies and families with an effective quality of life are a social resource.” [15] (p. 188). These are all solid reasons that support the adoption of this family model in EC, a model that has the family as a core dimension and whose ultimate goal is to promote a better quality of family life. In fact, Bhopti et al., stated that EC services should demonstrate “positive family outcomes annually” [12] (p. 192).

Family quality of life (FQoL), then, should be a relevant indicator of service quality [13]. The problem, however, is that there is no consensus in the definition of FQoL, nor it is easy to change the approach and purpose of some services [2]. One of the most accepted theorizations of the concept of FQoL argues that “family quality of life is a dynamic sense of well-being of the family, collectively and subjectively defined and informed by its members, in which individual and family-level needs interact” [16] (pp. 262). This idea is reinforced by the unified theory developed by Zuna et al., according to which “[systemic factors] directly impact individual and family-level supports, services, and practices. Individual-member concepts (i.e., demographics, characteristics) are direct predictors of FQoL and interact with individual—and family-level support, services, and practices to predict FQoL. Singly or combined, the model predictors result in a FQoL outcome that produces new family strengths, needs, and priorities that re-enter the model as new input resulting in a continuous feedback loop throughout the life cycle” [16] (pp. 269).

This integrative and multidimensional model does not make easy to assess FQoL. The current evaluation methods most recognized and used internationally are the International Family Quality of Life Project [17] and the Beach Center Family Quality of Life Scale [18]. Each one has a different operational definition of FQoL and each one identifies a different number of dimensions to focus on in the evaluation—nine in the former and five in the latter. At a national level, in Spain there is only one instrument to evaluate early care services—the Spanish Family Quality of Life Scale (FQoL-S) developed by Giné et al. [19]—which only covers until 18 years of age. The FQoL-S started including seven dimensions, but after a review of its psychometric properties in 2018, it was reduced to five dimensions. This scale was created and developed exclusively for population with intellectual disabilities. The limitations of this instrument do not allow us to know if the Spanish EC services are moving in the right direction. All of this limits us, while occupying our interest in how to know if our EC services are moving in a better direction.

The Spanish Federation of Associations of Early Care Professionals has already acknowledged that it is time to move towards a common early care model in Spain. They argued that early care must be recognized as a subjective right through a state law or regulation that includes all children from 0 to 6 years of age who have problems and developmental issues at some point in their development. This represents 10% of the child population of that age group, which means caring for 255,277 children in Spain in the Child Development and Early Care Centers (Centros de Desarrollo infantil y atención primaria, or CDIATs). 

Our systematic review was based on all the factors mentioned above together with the fact that “the evolutionary and vital circumstances of children and their families are changing and are different from those that characterize early childhood” [2]. Our main goal was to provide answers to the following research questions:

(1) What has been the conceptualization of the quality of life of families with a child who has a disability or developmental concerns between 0 and 6 years?
(2) What instruments of FQoL directed to population with disability and that have adequate psychometric properties exist for the child stage (0–6 years)?
(3) What are the main findings of the existing studies on FQoL in the 0–6 years stage?

## 2. Method

The search focused on three databases, Web of Science, Scopus and Eric, and it was based on the following keywords: “Family Quality of Life” OR “Quality of Family Life” (in English) and “Calidad de Vida Familiar” (in Spanish). We decided to exclude the term ‘disability’ in our search in order to enrich our understanding on FQoL, to cover any type or diagnosis of disability and to improve our current measurements on disability related FQoL. 

Our selection of the bibliographic materials was based on the following criteria:

(a) the empirical studies were based on studies with samples that included families of children with disabilities and/or developmental issues within the 0 to 6 years stage;
(b) the studies had been published after 1999, which is the year when the publications on FQL as a social construct and extension of the QoL of individuals with IDD started;
(c) the studies were written in English or Spanish, as these are the languages used in most of the publications on this topic and the languages mastered by the authors of this article;
(d) the studies were published in peer-review journals or as book chapters.

As for the exclusion criteria, we did not consider studies in which:

(a) disability was considered a disease, since that would have entailed to discard the systematic approach in favor of the rehabilitative medical model;
(b) FQoL was studied from an individual-based perspective (instead of the holistic model mentioned above which includes all the family members);
(c) FQoL was conceptualized from a rehabilitative medical perspective, since we are interested in disability from a psychosocial approach.

In order to ensure reliability, two independent researchers conducted the literature review and there was full agreement on their results. Besides, two separate searches were conducted in parallel: one focused on the scientific literature in English and another one focused on the scientific literature in Spanish.

## 3. Results

Our first bibliographic search retrieved a total of 1119 articles, 33 in Spanish and 1086 in English (see Table 1). From the 1119 articles, 493 were discarded as duplicate materials and seven more publications were incorporated from additional sources. The breakdown of these seven publications is as follows:

(1) four theoretical studies, which provide definitions of FQoL [16,20,21,22];
(2) the Family Quality of Life Survey (FQOLS-2006) [23] conducted by an international team of researchers;
(3) the study by García Grau et al. [24] published months after the searches were conducted;
(4) an empirical study [25] found as grey literature.

**Figure 1:**
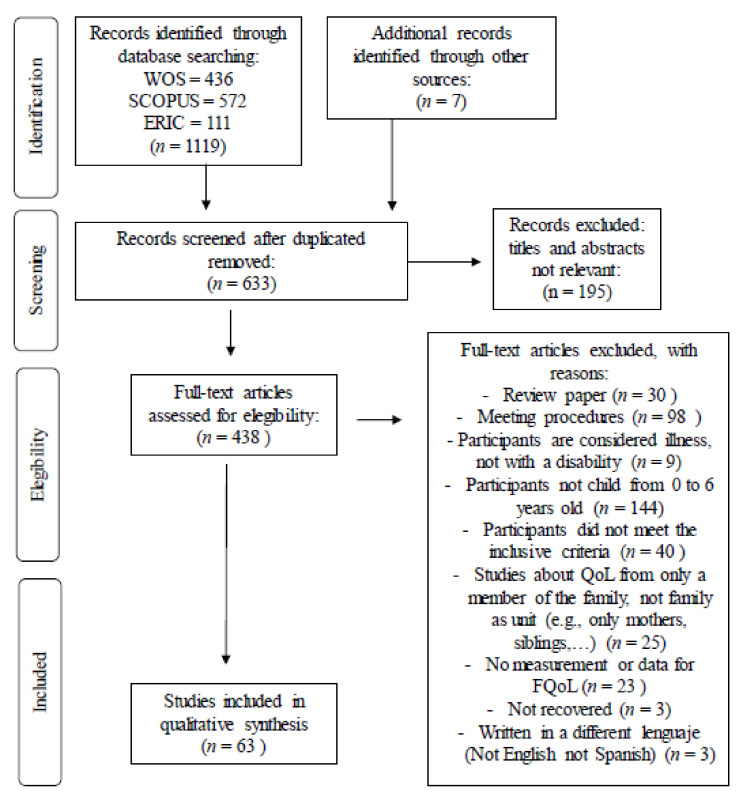
Flowchart of the selection process according to the recommendations of the Prisma statement. (Moher and Liberati [26]). Source: Own elaboration.

The implementation of the inclusion and exclusion criteria reduced the total selection of articles to 663 records. Of these, 195 studies were removed after reading the titles and abstracts. After reading the full text of the remaining 438 records, 375 articles were also discarded. The reasons for their exclusion are included in the flowchart below (see Figure 1). Thus, the final number of articles in which our systematic review is based is 63, from which 60 are written in English and three in Spanish.

The selected articles were analyzed and classified according to the three research questions mentioned above.

### 3.1. The Conceptualization of FQoL in the 0 to 6 Year Stage

Considering the 63 articles reviewed, two ways of addressing the conceptualization of FQoL have been identified: (1) how it has been theoretically defined (Table 2) and (2) how it has been measured or operationalized (Table 3). Hu et al. [27] called this second approach “functional” because it identifies the areas or domains of family life that are measured through scales or other instruments. This Systematic Review (SR) follows Hu’s functional approach.

#### 3.1.1. Theoretical Conceptualization

Being aware of the different conceptualizations of FQoL is an important step in the field of disability because “it is difficult to advance in any field if a definition of the concept or phenomenon studied is not commonly shared and if there is uncertainty about what, in the first place, is supposed to be measured” [31] (p. 19). The existing definitions of FQoL are included in Table 2.

From a chronological point of view, the first definition was formulated in 2000 by Turnbull et al. [22] cited by Park et al. [28]. This definition includes the perceptions of family members, and for this reason some authors call it “subjective conceptualization” [32,33]. The influence of this conceptualization in the FQoL literature is relevant [34,35,36,37].

Four years later, in 2004, Brown and Brown [20] identified three components in FQoL. According to them, families experience quality of life when they (a) strive to achieve what they want; (b) are satisfied with what they have achieved; (c) feel empowered to lead the life they desire. Ten years later, these components were also included in another study by the same authors, which highlights how the FQoL construct “changes a little over time in response to our understanding of other related concepts, changing social values and norms and cultural and environmental conditions” [21] (pp. 2195).

In 2010, Zuna et al. [16] proposed a definition that highlights the dynamic meaning of the construct. This definition has been cited by 15 of the articles selected in this study [6,33,37,38,39,40,41,42,43,44,45,46,47,48,49].

#### 3.1.2. Functional Conceptualization

From the operational or functional point of view, the FQoL is understood as “a global outcome of services” [50] (pp. 204). At first, the conceptualization of the FQoL was based on the one used in the field of the individual QoL, which led to the consideration of the FQoL as a multidimensional construct. Indeed, researchers of FQoL have focused on identifying the different domains that constitute the FQoL construct through the creation and validation of measurement instruments.

Table 3 shows the domains that constitute the instruments created to implement the FQoL construct, including the name and number of domains in each proposal. There are six functional conceptualizations so far. Although the number of domains in each of the proposals varies (from 9 domains by the FQOL Survey-2006 [17] to 3 of the FEIQoL [24]), the following domain names are repeated: family, supports and financial.

### 3.2. Instruments that Measure the FQoL in the 0 to 6 Years Stage

The results of the bibliographic search conducted for this review show that the majority of studies focused on the 0 to 6 years stage have used international FQoL scales, which were designed for all ages, such as the Beach Center FQoL Scale [18], the FQOL Survey-2006 [17] and FQoL-S [19]. Table 4 only includes the instruments designed to be applied in children between 0 and 6 years of age.

There are three instruments designed to be applied in EC: (1) The Autism Family Experience Questionnaire (AFEQ) [29] for families with children with autism; (2) ITP-Child Quality-of-Life Questionnaire [30] for children with immune thrombopenic purpura; and (3) the adaptation of the Family Quality of Life (FaQoL) by McWilliam et Casey to the Spanish context (FEIQoL) [24] for children with all types of disabilities.

In the search for instruments to measure the FQoL and in addition to the instruments mentioned in Table 3 and Table 4, we also identified 10 studies whose main objective is the development, adaptation and/or validation of the FQoL scales (see Table 5). 

As we can see, these 10 studies refer to the most international FQoL scales.

### 3.3. Main Results on FQoL in the 0- to 6-Year Stage

In response to the third research question about the main finding of the studies on FQoL in the 0–6 years, the studies applied to this age group have been divided into two tables. The first table (Table 6) classifies 22 studies that describe, compare or relate QoL of families with young children with disabilities with other variables. The second table (Table 7) shows the studies that focus on the variables that predict FQoL. 

The breakdown of the results found in the studies included in Table 6 is as follows:

(a) Ten articles explicitly focus on describing the FQoL in the population of their respective countries. Spain [6,44,62] has three studies; Israel [32,60] has two, and Australia [36], Brasil [63], Colombia [59] and Malaysia [61] have one each.
(b) Two articles [64,65] relate FQoL to parental stress in families with children with autism.
(c) Six articles relate the FQoL to a specific type of disability. Brown et al. [66], for instance, compared the QoL of three types of families: families with a child with Down syndrome, families with a child with autism spectrum disorders, and families with none of their members having a disability. The other five articles focus on specific disabilities, namely deafness [48], intellectual disability [32,67], autism [37] and rare metabolic diseases [68].
(d) Two articles adopt an ethnic perspective. Algood et al. [70] address the issue of inequity in the care of African-American families compared to other ethnicities, while Holloway et al. [69] study QoL in California among Latino and non-Latino families.
(e) Two articles relate the FQoL to perspectives of some of the family members. Moyson and Roeyers [72] investigated the FQoL from the perspective of the siblings of the person with disabilities. Wang et al., determined “whether mothers and fathers similarly view the conceptual model of FQoL embodied in one measure” [71] (pp. 977). This study shows that there are no significant differences between the perceptions of fathers and mothers.
(f) Two studies investigate how families describe the supports and services they receive [24,71].

Following the proposal of Zuna et al. [8], the 21 studies analyzed in Table 7 identify the following components: (a) systemic concepts; (b) performance concepts; (c) individual-member concepts; and (d) family-unit concepts.

First, the systemic concept is integrated by three categories: (a1) systems; (a2) policies; and (a3) programs. Regarding the first two categories, no study has been identified. As for the third category (“programs”), Hielkema et al. [74] studied the effectiveness of “Coping with and Caring for infants with special needs (COPCA)”, a family-focused program applied to 43 families with young children at high risk of cerebral palsy. The results related to the group that received COPCA show that the FQoL improved over time.

Second, the performance concepts focus on the following three categories: (b1) Services; (b2) Supports and (b3) Practices. Seven studies focus on services (b1), and all of them show that the services received by families with young children with disabilities favor FQoL [7,43,46,49,50,75,76]. Balcells-Balcells et al. [77] and Samuel et al. [75] identify an increase in parental satisfaction based on the information received by the services. Taub and Werner [49] found that both religious and secular families are satisfied with the support received from the spiritual community and the social services, respectively. Eight studies focus on supports (b2). The support of professionals to families has received significant attention, and it has become one of the strongest predictors of FQoL [58,78,79]. Emotional support is better considered than practical support from both services and other informal aids [35,41,73,76,80]. In fact, the study by Meral et al. [80] reveals that emotional support is the most important factor for the respondents. Svavarsdottir and Tryggvadottir [81], on the other hand, focus on the predictive nature of family support. Finally, considering the category of practices (b3), Davis and Gavidia Payine [79] recognize the value of the family-centered model as a safeguard for the needs of each family.

Third, the individual-member concepts integrates the following three categories: individual characteristics (c1), demographic aspects (c2) and beliefs (c3). In the individual characteristics category (c1), six articles were related to parental stress. The majority of them focus on studying how the support [33,79,81,82] and the information received [79,81] are predictive factors of the decrease in parental stress and, consequently, of the increase in FQoL [78]. Wang et al. [83] indicate that the efforts that parents with children with disabilities make in defending their kids generates considerable stress in them. Boehm et al. [84] investigate the relationship between the parents’ religiosity and the improvement of the FQoL.

**Table 7:** Predictive studies of the FQoL.

Concepts of FQoL Theory (Zuna et al. [8])		Authors (Year) (Chronological Order)
Systemic concepts	Systems	No records have been identified
	Policies	No records have been identified
	Programs	Hielkema et al., 2019, [74]
Performance concepts	Services	Balcells-Balcells et al., 2019 [77]; Epley et al., 2011 [50]; Eskow et al., 2011 [43]; Kyzar et al., 2016 [46]; Samuel et al., 2012 [75]; Summers et al., 2007 [76]; Taub and Werner 2016 [49];
	Supports	Boehm et al., 2019 [84]; Cohen et al., 2014 [41]; Davis and Gavidia Payne, 2009 [79]; Hsiao et al., 2017 [78]; Kyzar et al., 2016 [46]; Kyzar et al., 2018 [85]; Meral et al., 2013 [80]; Samuel et al., 2011 [36]; Svavarsdottir and Tryggvadottir, 2019 [81]; Taub and Werner, 2016 [49]
	Practices	Davis and Gavidia Payne, 2009 [79]
Individual-member concepts	Individual characteristics	Boehm et al., 2019 [84]; Davis and Gavidia Payne, 2009, [79]; Hsiao et al., 2017 [78]; Hsiao 2018, [82]; Levinger et al., 2018, [86]; Meral et al., 2013 [80]; Vanderkerken et al., 2018 [33]; Wang et al., 2004 [83];
	Demographic aspects	Meral et al., 2013 [80]; Cohen et al., 2014 [41]; Hsiao 2018 [82]; Kyzar et al., 2018 [85]; Levinger et al., 2018 [86]; Vanderkerken et al., 2018 [33]; Boehm et al., 2019 [84]
	Beliefs	Svavarsdottir and Tryggvadottir, 2019 [81]
Family-unit concepts	Characteristics of the family	Boehm et al., 2019 [84]; Cohen et al., 2014 [41]; Davis and Gavidia Payne, 2009 [79]; Hsiao 2018 [82]; Hielkema et al., 2019 [74]; Kyzar et al., 2018; Schlebusch et al., 2016 [87]; Taub and Werner 2016 [49]; [85]; Wang et al., 2004 [83]
	Family dynamics	Schlebusch et al., 2016 [87]; Levinger et al., 2018; [86]; Vanderkerken et al., 2018 [33].

In the demographic aspects category (c2), nine articles consider as predictive variables for FQoL the following factors: the age of the child with a disability [41,80,83,85], the gender of the child [24,39,82], the type of disability [31,80,84], the degree of disability [80,81], the number of siblings [58,81], and the marital status of the parents [44].

Finally, in the category focused on beliefs, (c3), Svavarsdottir, and Tryggvadottir [81] conclude that the beliefs of the parents regarding the severe illness of the child significantly predicted the FQoL.

The last factor of the proposal by Zuna et al. [8] is the family-unit concepts, which integrates two categories: family unit (d1) and family dynamics (d2). In the section on the family unit (d1), 9 studies consider the predictive factors of FQoL. The results show that for the most part two-parent families enjoy better FQoL than single-parent families [62,82]; family income is predictive of better FQoL [79,82,87]; and belonging to one or another ethnic group [41,82], being part of a religious community [49] or having a spiritual faith [84] also determines the level of satisfaction of FQoL. Regarding family dynamics (d2), the study by Vanderkerken et al. [33] addresses the so-called homeostatic control and suggests asking the opinions of all family members about the FQoL.

## 4. Discussion

The discussion is developed in the order of the research questions stated at the outset of this review. Regarding the first question based on the FQoL conceptualization, there is an incipient holistic approach, but the functional conceptualization still prevails. We will analyze our findings in relation to two previous SRs focused on the conceptualization of the FQoL [88] and on the FQoL measurement tools, respectively. Both SRs found that the FQoL scales lack a theoretical framework and a definition of this construct. Most researchers who published their studies after these two SRs followed the functional conceptualization approach. For instance, in a theoretical study which integrated the perspectives of three authors from three different research teams—Schippers, Zuna and Brown—it was argued that “from the QoL conceptual development and research to date, we have a strong sense that it is usually a number of variables across a variety of life areas working in an interrelated fashion that are essential to improving QoL for individuals and families” [89] (pp. 151). Although these authors emphasize the interrelated dimension of the different variables considered, many studies that explicitly refer to the holistic theory developed by Zuna only identify the different domains of family life but do not integrate them in their studies. 

Our SR has shown that the empirical studies arrive at different, and sometimes discordant, results. The discrepancies are based on how researchers have conceptualized FQoL, the number of the domains considered to evaluate FQoL, the methodology used (i.e., quantitative or mixed) and/or how many members of family have participated.

We agree with Boelsma et al. [90] on the need of taking into consideration the dynamic interaction between the individual and the family domains in the FQoL research. The fact that many studies refer to the holistic approach of FQoL by Zuna et al. [16] shows that this holistic understanding is becoming more and more solid in the conceptualization of the FQoL. What Zuna’s definition adds to the rest of the existing definitions (see Table 2) is the dynamic character, referring to the sense of well-being and the collective dimension behind the definition and evaluation of the FQoL construct, since it is based on the interaction between individual and family needs [91], and among services, supports and practices. 

The complexity underpinning this holistic approach does not reside in its multidimensionality but rather in its dynamic aspect. To study a dynamic reality entails understanding it from its capacity to face changes. Our understanding of this dynamic sense of family well-being follows Bhopti et al. [12] who emphasize how family well-being may change depending on significant events in the life of the family. 

Considering the second research question, based on the FQoL assessment instruments, there are no scales developed specifically for the 0–6-year stage, nor do the developed scales address all disabilities and/or developmental concerns. The FQoL-S by Giné et al. [19] covers up to 18 years of age. The FEIQoL of García Grau et al. [24] focuses on the measurement of early care services [44] and puts emphasis on the factor “child functioning” [38]. These same authors [24] recognise not having conceptualized the EI as extensively as they did the other scales. This acknowledgment questions the solidity of the theoretical framework on which their conceptualization is based.

The FEIQoL scale takes into account both the dynamic sense of the FQoL and the fact that the dynamic sense can change in the different stages of the family life cycle. However, in the studies of these authors, the dynamic concepts mentioned by Zuna are not explicitly present in the FEIQoL, except for the notion of family routines.

Finally, regarding the third research question about the findings, this SR has found that research on FQoL has mainly focused on issues related to disability and chronic illnesses in children from 0 to 6 years of age, even though the FQoL is construct that can be applied to a wider typology of families (families with a child who has not been diagnosed). 

Our analysis of the descriptive, comparative and correlational studies identified reveals that most of them focus on the individual concepts of the unified theory of FQoL by Zuna et al. [16]. Specifically, most of them focus on the characteristics of the individual, the characteristics of the child, and on demographic aspects. 

Among the predictive studies of the FQoL, there is more interest in the studies related to supports and services, individual categories, and categories related to the family unit than in the studies that focus on systemic aspects and family dynamics. Considering the former, it is important to highlight that there are no conclusive results in relation to the variables indicated, in particular to the variables related to the age and the disability of the children [32,44,67]. There is only partial agreement in the studies that show how the FQoL of the families with children with disability in the first two years of age is less than the FQoL of the families with children with disability from three years of age on [44,62]. A wider agreement is reached among the studies that show that the greater the severity of the child’s disability, the lower the degree of QoL in families [6,79,86,87]. We noticed that although quantitative studies predominate, there is an increase in others that incorporate qualitative [42,63,72] or mixed methodology [35,66,67,82]. We also identified the need of match the methods used with the ethical dimensions of the research.

Qualitative research studies are needed [42] if we want to evaluate family outcomes related to experiences “that can only be explained by considering the perceptions of the family members themselves, because ultimately it is these subjective perceptions that determine the individual’s approach to life and how satisfied they are with life” [7] (pp. 17). In this regard, some researchers adapt their methodologies to the participants in their research. Van Heumen and Schippers [92], for instance, use the Photovoice methodology to allow the family members to speak about the images that are significant to them.

Finally, a few words on the ethical aspects of research in FQoL. As we know, one of the ethical requirements of research refers to the coherence between the objectives and intentions of the researchers and the results obtained. From the beginning, scholars doing research on FQoL identified a problem that presented both a practical and an ethical aspect. Poston et al., included in their study the opinions of various authors regarding the need to consider the perceptions of all family members. Yet, they soon identified a practical problem—despite trying to involve the other members, usually only the mother participated. From an ethical perspective, these authors consider this fact to be crucial to evaluate the realiability of the results [93]. In 2006, Wang et al., published a study in which they proposed to verify if both parents understood the QoL of their family in a similar way [71]. Both the fathers and the mothers responded in a similar way, which turned out to be promising for the use of the scales in the case that only one of the parents participated. This conclusion prompts some researchers to include only one of the parents in the research [6,8,10,50,63,69]. Although Vanderkerken et al., argued that the perceptions of fathers did not differ significantly from those of mothers in a study in which they had a broad representation of family members [33], it is still important to investigate the impact that the participation of one or more family members has in the FQoL.

There are numerous studies that explicitly address the limitations of not taking into account more than one of the parents, which shows that there is an ethical concern in this regard [35,39,42,43,45,46,53,54,55,60,75,82,84]. Gardiner and Iarocci [91] indicate that in the future, research on the FQoL should include the voices of the different members of the family. Hu et al., establish a connection between the holistic nature of the FQoL and the method used. To solve this problem, Brown et al., propose that one of the parents responds on behalf of the rest of the family [66], who would thus be represented by him or her [74,87,94]. However, as Giné et al., note, there is no way to ensure that this instruction of responding on behalf of the family has been followed by the family representative [58]. Feigin et al. [95] understand that the participation of brothers and sisters of the child with disabilities, in addition to the parents, is a requirement of the systemic nature of the family.

## 5. Implications

This SR clearly shows that the state of the art in the research of FQoL points to the ecological, systemic and inclusive vision of the family and, therefore, of the FQoL in the field of EC and disability. The inclusion of studies without the term “disabilities” as a keyword in this SR has contributed to including enriching studies on this topic and to cover any type or diagnosis of disability. This inclusive perspective is an invitation to all services and institutions to direct their attention to the dynamic interaction of personal and collective needs.

As this SR shows, there is a need to develop the emerging holistic conceptualization through mixed research and to be open to methodologies that overcome the limitations mentioned above. We also need to create instruments of measurement of the FQoL that are specific to this stage of life and family cycle from a systemic perspective.

## 6. Limitations

We were not able to get access to three studies found in the databases which were included in this review based on the title and/or the abstract [96,97,98].

## 7. Conclusions

Empirical studies of the QoL of families with a child with a disability or developmental concerns show a certain inertia of functional conceptualization. Yet, an incipient holistic conceptualization has also been noted. Among the selected articles, three instruments have been identified to measure the QoL of families with young children in the age range 0–6 years: (1) The Autism Family Experience Questionnaire (AFEQ) [29]; (2) ITP-Child Quality-of-Life Questionnaire (Barnard et al. [30] and (3) Family Early Intervention Quality of Life (FEIQoL) [24]. These instruments however do not refer to all disabilities or do not have a holistic approach. Considering the main predictor variables studied here—the age of the child and the type of disability—there are no unanimous or conclusive results. After the investigation, the SR has become a State of the Art of the FQoL research, since it identifies the last contributions in conceptualization, and the epistemological and methodological deficiencies on the studies of FQoL. This SR identifies two key areas for future research: to deepen the understanding of the dynamic interactions of family relationships and to understand the ethical requirement of having the methods used to approach the FQoL respect the holistic nature of the research.

## Figures and Tables

**Table 1:** Search procedures for English and Spanish publication.

Plataform	Results	Search	Languages
Scopus	563	“Family Quality of Life” OR “Quality of Family Life”	English
	9	“Calidad de Vida Familiar”	Spanish
WOS	416	“Family Quality of Life” OR “Quality of Family Life”	English
	20	“Calidad de Vida Familiar”	Spanish
Eric	107	“Family Quality of Life” OR “Quality of Family Life”	English
	4	“Calidad de Vida Familiar”	Spanish
Total	1119		

**Table 2:** Theoretical conceptualization of FQoL.

Definitions of Family Quality of Life	Articles
Conditions where the family’s needs are met, and family members enjoy their life together as a family and have the chance to do things which are important to them.	Turnbull et al. [22] Cited by Park et al. [28] (pp. 368)
It can be said that families experience a satisfactory quality of family life when: (a) they achieve what families around the world, and they in particular, strive to achieve; (b) they are satisfied with what families around the world, and they in particular, have achieved; (c) they feel empowered to live the lives they wish to live.	Brown and Brown [20] (pp. 32)
Family quality of life is a dynamic sense of well-being of the family, collectively and subjectively defined and informed by its members, in which individual and family-level needs interact.	Zuna et al. [16] (pp. 262)
Family quality of life is concerned with the degree to which individuals experience their own quality of life within the family context, as well as with how the family as a whole has opportunities to pursue its important possibilities and achieve its goals in the community and the society of which it is a part.	Brown and Brown [21] (pp. 2195)

**Table 3:** Conceptualization of FQoL across six researcher groups.

Beach Center FQoL scale (Hoffman et al. [18]) 5 Domains	FQoLsurvey-2006 International Project (Brown et al. [17]) 9 Domains	FQoL-S (Giné et al. [19]) 7 Domains	The Autism Family Experience Questionnaire (AFEQ) (Leadbiter et al. [29]) 4 Domains	ITP-Child Quality-of-Life Questionnaire (Barnard et al. [30]) 5 Domains	Feiqol-Family Early Intervention Quality of Life (Garcia-Grau et al. [24]) 3 Factors
1. Family Interaction 2. Parenting 3. Emotional Well-being 4. Physical-Well-being material 5. Disability Related Support	1. Health 2. Finances 3. Family Relationships 4. Informal Support 5. Service Support 6. Influence of values 7. Career path 8. Leisure and free time 9. Community	1. Emotional well-being 2. Family Interaction 3. Health 4. Final well-being 5. Organization and parenting skills 6. Accomodation of the family 7. Social Inclusion and Participation	1. Parents 2. Family 3. Child development 4. Child symptoms	1. Treatment side effect-related 2. Intervention related 3. Disease-related 4.Activity-related 5. Family-related	1. Family Relationships 2. Access to Information and Services 3. Child Functioning

**Table 4:** FQoL instruments in the 0- to 6-year stage.

Instruments and Authors	Respond	Domains	Dimensions	Number of Items and Reliability
The Autism Family Experience Questionnaire (AFEQ) (Leadbiter et al. [29])	Parents	*4* domains: (1) Parents; (2)Family; (3) Child development; (4) Child symptoms	Likert Scale of frequency 1 to 5 points (with “not applicable” option)	56 items
ITP-Child Quality-of-Life Questionnaire (Barnard et al. [30])	Parents	*5* domains: (1) treatment side effect-related; (2) intervention related; (3) disease-related; (4) activity-related; and (5) family-related	Likert scale of frequency and importance 1 to 5 points	26 items
Family Early Intervention Quality of Life (FEIQoL) García Grau et al. [24] *		*3* Factors: (1) Family Relationships; (2) Access to Information and Services; (3) Child Functioning	Likert Scale 1 to 5 points of “poor” to “excellent”	40 items

* The Family Early Intervention Quality of Life (FEIQoL) García Grau et al. [24] is the Spanish version of the original instrument developed by McWilliam and Casey 2013 (unpublished) [51].

**Table 5:** Studies that adapt, develop or validate the FQoL scales.

Scales	Development, Validation or Adaptation Studies	Country
Beach Center FQoL Scale (Hoffman et al., 2006) [18]	Balcells-Balcells et al., 2011 [39]; Verdugo et al., 2005 [52]	Spain
	Chiu et al., 2017 [40]; Chiu et al., 2017 [53]	Hong Kong
	Waschl et al., 2019 [54]	Singapore
	Bello-Escamilla et al., 2017 [55];	Chile
	Rivard et al., 2017 [56]	Canada
FQOL Survey-2006 (Brown et al., 2006) [17]	Perry e Isaacs 2015 [57]; Samuel et al., 2016 [15]; Samuel et al., 2018 [58]	USA

**Table 6:** Descriptive, comparative or correlational studies.

Theme	Authors (Year) (Chronological Order)
Population	Córdoba et al., 2008 [59]; Neikrug et al., 2011 [60]; Clark et al., 2012 [61]; Rillotta et al., 2012 [35]; Giné et al., 2015 [62]; Mas et al., 2016 [6]; Schertz et al., 2016 [32]; García Grau et al., 2018 [44]; Rodrigues et al., 2018 [63].
Maternal Outcomes	McStay et al., 2014 [64,65].
Type of disability	Brown et al., 2006 [66]; Jackson et al., 2010 [48]; Schertz et al., 2016 [32]; Tait et al., 2016 [67]; Schlebusch et al., 2017 [37]; Tejada-Ortigosa et al., 2019 [68].
Ethnic perspective	Holloway et al., 2014 [69]; Algood and Davis, 2019 [70].
Attention to participants	Wang et al., 2006 [71]; Moyson and Roeyers, 2012 [72].
Supports	Steel et al., 2011 [73]; Escorcia-Mora et al., 2018 [25].
